# 

**Published:** 1885-08

**Authors:** 


CONTENTS

Vol. 32 No. 8

| | PAGE. | | PAGE. |
|---|---|---|---|
| How to Eat Wisely | 1 | The Delusion of Growing Fat | 10 |
| How to Avoid Colds | 2 | For Stutterers | 10 |
| Air, Sunshine and Health | 3 | Catarrh | 11 |
| Swine Disease and Disinfectants | 4 | Physiological Aphorisms | 12 |
| The Approach of Age | 5 | Inoculating for Cholera | 14 |
| Curing Rheumatism with Celery | 5 | Taking Medicine | 14 |
| How Ladies Should Ride Horseback | 6 | Checking Perspiration | 15 |
| Staining Room Floors | 7 | The Teeth | 17 |
| Animal Language | 7 | Pain | 18 |
| | | Poisons | 20 |

